# Supplementary material for: Modulation of metastable ensemble dynamics explains optimal coding at moderate arousal in auditory cortex
Source: bioRxiv. 2024 Apr 8:2024.04.04.588209. Preprint. [Version 2] doi: 10.1101/2024.04.04.588209 (PMC11014582; doi:10.1101/2024.04.04.588209)
Supplement: Supplement 1 [file media-1.pdf]

Supplementary Information

Parameter	Description	Value
N_E	number of E cells	1600
N_I	number of I cells	400
τ_{m}^E	membrane time constant of E cells	20 ms
τ_{m}^I	membrane time constant of I cells	20 ms
τ_{syn}^E	E synaptic time constant	5 ms
τ_{syn}^I	I synaptic time constant	5 ms
τ_{ref}^E	refractory period of E cells	5 ms
τ_{ref}^I	refractory period of I cells	5 ms
V_{t}^E	threshold potential of E cells	1.5 mV
V_{t}^I	threshold potential of I cells	0.75 mV
V_{r}^I	reset potential of I cells	0 mV
V_{r}^I	reset potential of I cells	0 mV
p_{EE}	E-to-E connectivity fraction	0.2
p_{IE}	E-to-I connectivity fraction	0.5
p_{EI}	E-to-I connectivity fraction	0.5
p_{II}	E-to-I connectivity fraction	0.5
J_{EE}	uniform E-to-E synaptic strength	$0.63/\sqrt{N}$ mV
J_{IE}	uniform E-to-I synaptic strength	$0.63/\sqrt{N}$ mV
J_{EI}	uniform E-to-I synaptic strength	$1.9/\sqrt{N}$ mV
J_{II}	uniform E-to-I synaptic strength	$3.8/\sqrt{N}$ mV
p	number of E and I clusters	18
f_E	fraction of E cells/cluster	0.05
f_I	fraction of I cells/cluster	0.05
J_{EE}^+	within-cluster E-to-E synaptic strength	$15.75 \times J_{EE}$ mV
J_{IE}^+	within-cluster E-to-I synaptic strength	$5.45 \times J_{IE}$ mV
J_{EI}^+	within-cluster E-to-I synaptic strength	$6.25 \times J_{EI}$ mV
J_{II}^+	within-cluster E-to-I synaptic strength	$5.0 \times J_{II}$ mV
C_{ext}^{EE}	number of external synapses to E cells	320
C_{ext}^{IE}	number of external synapses to I cells	320
J_{ext}^{EE}	external E-to-E synaptic strength	$2.3/\sqrt{N}$ mV
J_{ext}^{IE}	external E-to-I synaptic strength	$2.3/\sqrt{N}$ mV
ν_o^E	baseline external rate to E cells	7 spks/s
ν_o^I	baseline external rate to I cells	7 spks/s
A_{stim}^E	relative stimulus amplitude for E cells	0.05
A_{stim}^I	relative stimulus amplitude for I cells	0
t_{stim}	stimulus onset time	1 s
τ_r	stimulus rise time	75 ms
τ_d	stimulus decay time	100 ms
Δ_M^E	strength of mean input modulation for E cells	variable
Δ_M^I	strength of mean input modulation for I cells	variable
Δ_H^E	strength of input heterogeneity across E cells	variable
Δ_H^I	strength of input heterogeneity across I cells	0

TABLE S1. **Parameter values for the spiking circuit model.**

Parameter	Description	Value
N_E	number of E cells	640
N_I	number of I cells	160
$\tau_{\rm m}^{E}$	membrane time constant of E cells	20 ms
$\tau_{\rm m}^{I}$	membrane time constant of I cells	20 ms
$\tau^{\rm syn}$	synaptic time constant	5 ms
$\tau_{\rm ref}^{E}$	refractory period of E cells	5 ms
$\tau_{\rm ref}^{I}$	refractory period of I cells	5 ms
$V_{\rm t}^{E}$	threshold potential of E cells	4.86 mV
$V_{\rm t}^{I}$	threshold potential of I cells	5.98 mV
$V_{\rm r}^{I}$	reset potential of I cells	0 mV
$V_{\rm r}^{I}$	reset potential of I cells	0 mV
p_{EE}	E-to-E connectivity fraction	0.2
p_{IE}	E-to-I connectivity fraction	0.5
p_{EI}	E-to-I connectivity fraction	0.5
p_{II}	E-to-I connectivity fraction	0.5
J_{EE}	uniform E-to-E synaptic strength	$0.8/\sqrt{N}$ mV
J_{IE}	uniform E-to-I synaptic strength	$2.5/\sqrt{N}$ mV
J_{EI}	uniform E-to-I synaptic strength	$10.6/\sqrt{N}$ mV
J_{II}	uniform E-to-I synaptic strength	$9.7/\sqrt{N}$ mV
p	number of E and I clusters	2
f_E	fraction of E cells/cluster	0.125
f_I	fraction of I cells/cluster	0
J_{EE}^{+}	within-cluster E-to-E synaptic strength	$20 \times J_{EE}$ mV
J_{IE}^{+}	within-cluster E-to-I synaptic strength	$1 \times J_{IE}$ mV
J_{EI}^{+}	within-cluster E-to-I synaptic strength	$1 \times J_{EI}$ mV
J_{II}^{+}	within-cluster E-to-I synaptic strength	$1 \times J_{II}$ mV
$C_{\rm ext}^{EE}$	number of external synapses to E cells	128
$C_{\rm ext}^{IE}$	number of external synapses to I cells	128
$J_{\rm ext}^{EE}$	external E-to-E synaptic strength	$12.9/\sqrt{N}$ mV
$J_{\rm ext}^{IE}$	external E-to-I synaptic strength	$14.5/\sqrt{N}$ mV
ν_o^{E}	baseline external rate to E cells	7 spks/s
ν_o^{I}	baseline external rate to I cells	7 spks/s
Δ_H^{E}	strength of input heterogeneity across E cells	$[0, 0.275]$
Δ_H^{I}	strength of input heterogeneity across I cells	0

TABLE S2. **Parameter values for the reduced 2-cluster circuit model.**

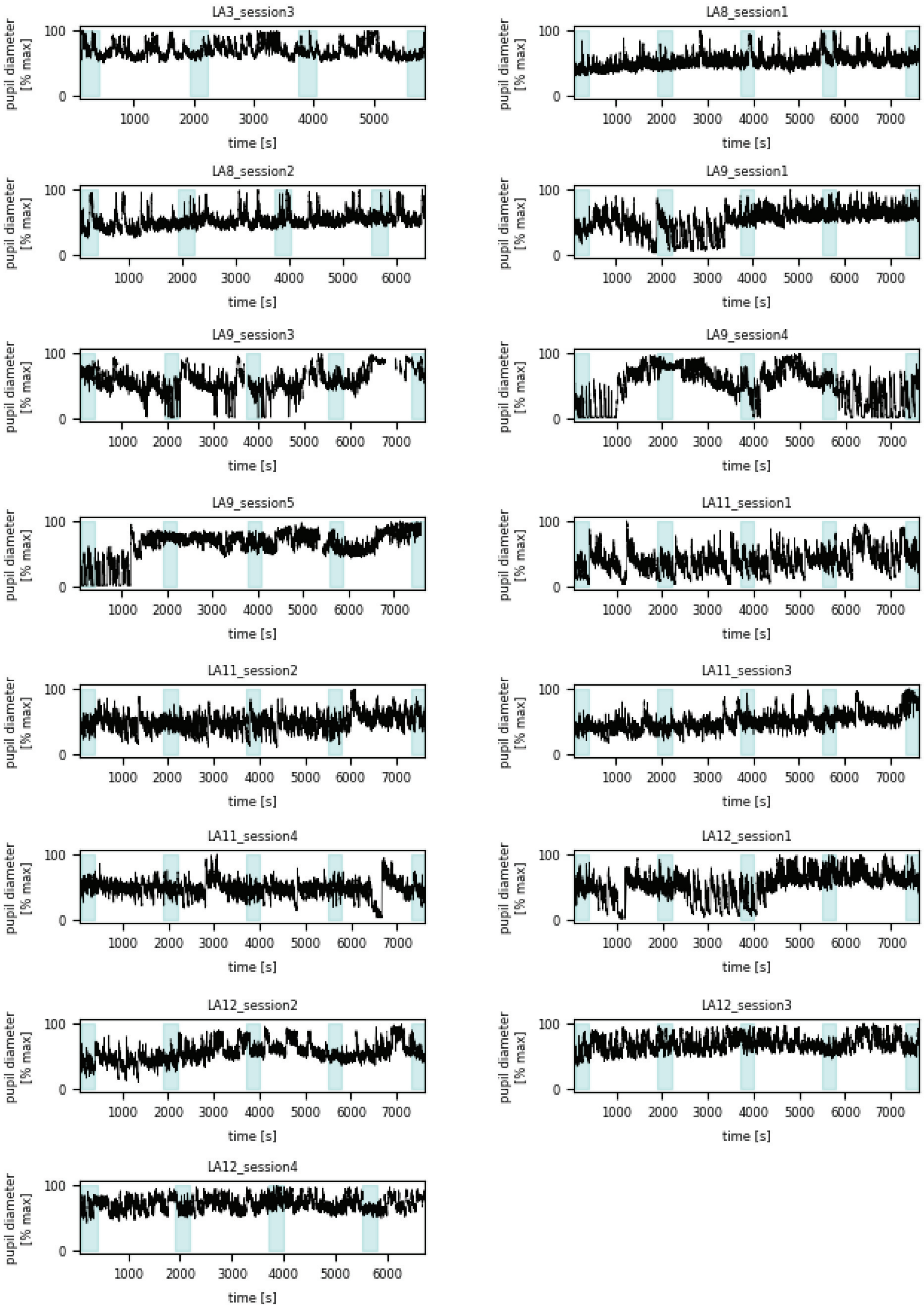

FIG. S1. Pupil diameter trace for each session. Light green areas indicate time segments during which no stimuli were presented (“spontaneous periods”) and white areas indicate segments during which pure tones were presented (“evoked periods”).

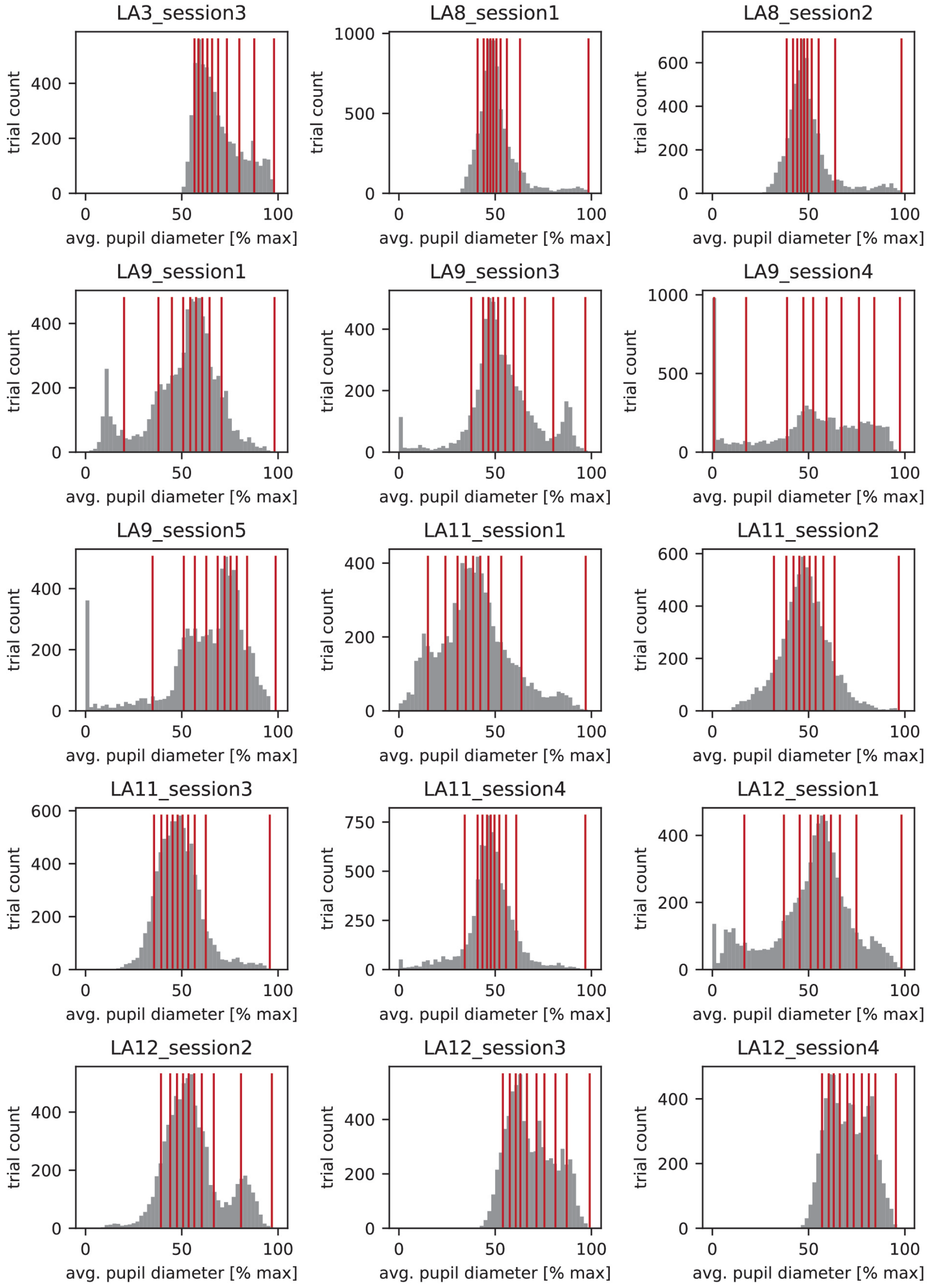

FIG. S2. Histogram of the pre-stimulus pupil diameter (average across 100 ms period before stimulus onset) in each session. Red lines indicate the deciles of the distribution.

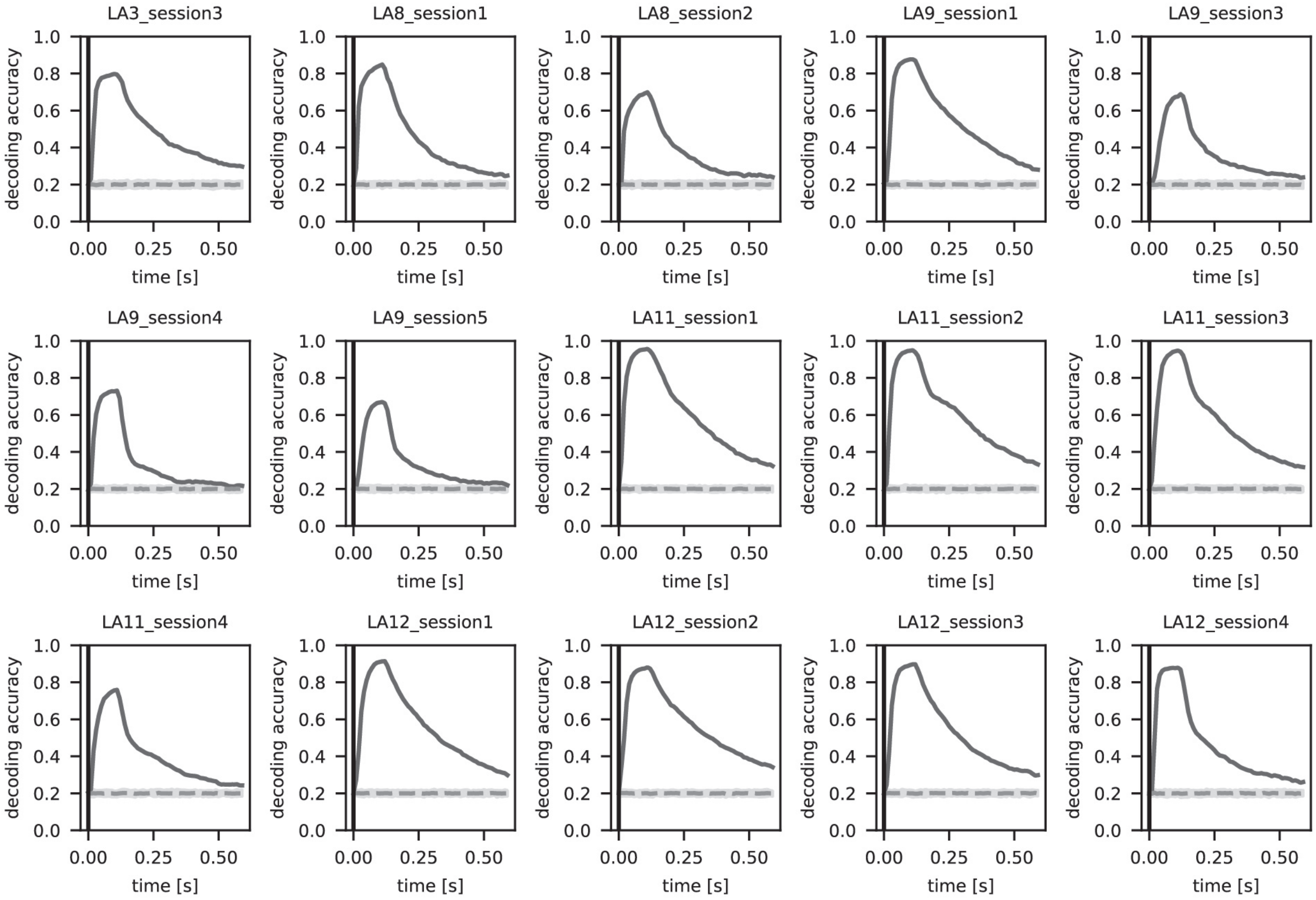

FIG. S3. Overall decoding accuracy *vs.* time relative to stimulus onset for each experimental session. In each panel, the vertical bar denotes the time of stimulus onset and the dark gray curve shows the average cross-validated time-course of the decoding performance; all trials (regardless of pupil diameter) were used to determine the overall decoding accuracy. The dashed gray line shows the mean of the shuffled accuracy distribution, and the light gray area denotes the 5th to 95th percentile range of the shuffled distribution. See Sec. IV C for methodological details.

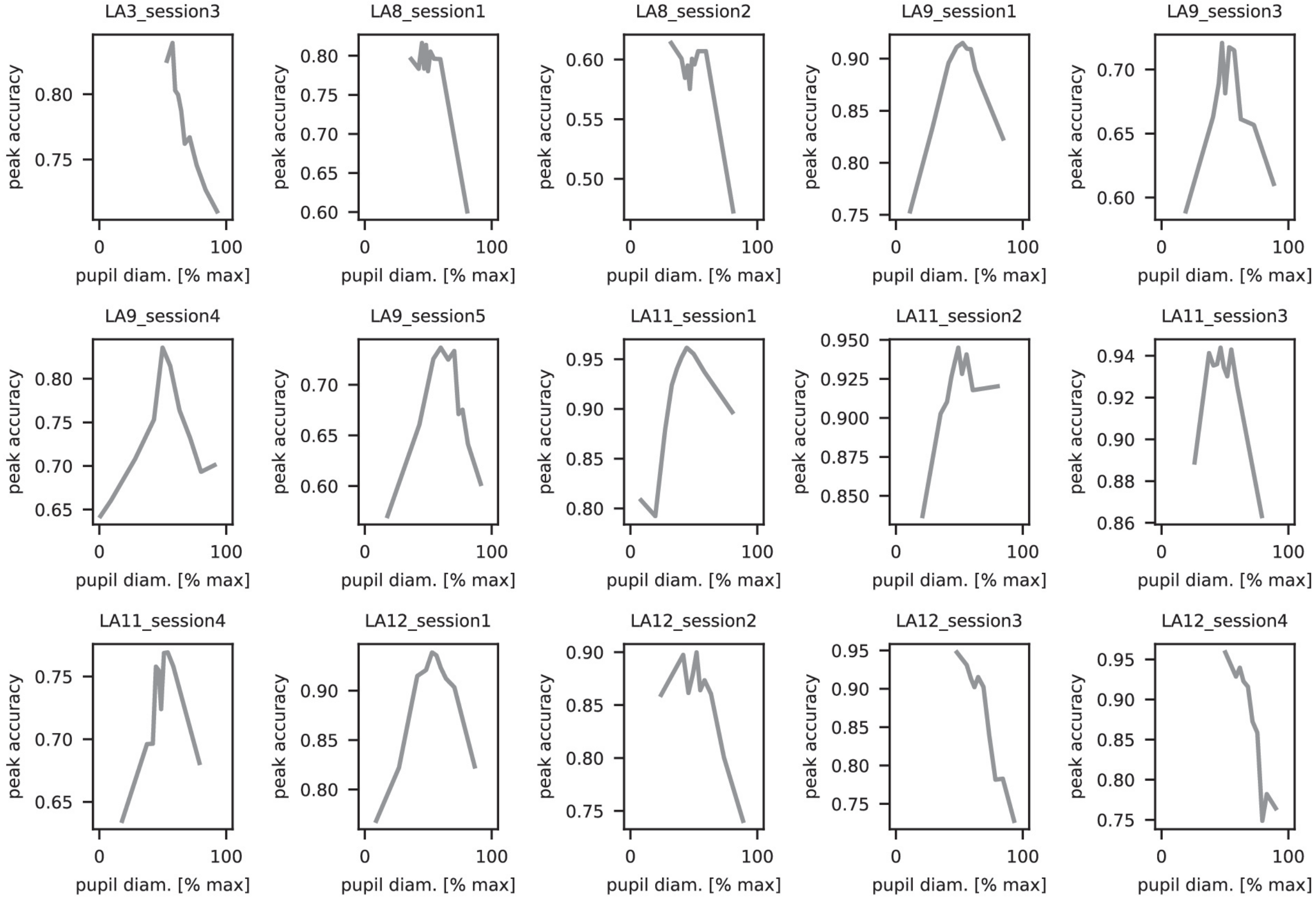

FIG. S4. Peak decoding accuracy *vs.* pupil diameter for all experimental sessions. In most recordings that achieved a broad range of arousal states, the decoding performance follows an inverted-U relationship with the extent of pupil dilation. For the remainder of sessions, in which only low-to-intermediate or intermediate-to-high diameters were thoroughly sampled, the corresponding upward or downward sloping portions of the curve are apparent (e.g., LA12_session3. See Sec. IV C for methodological details.

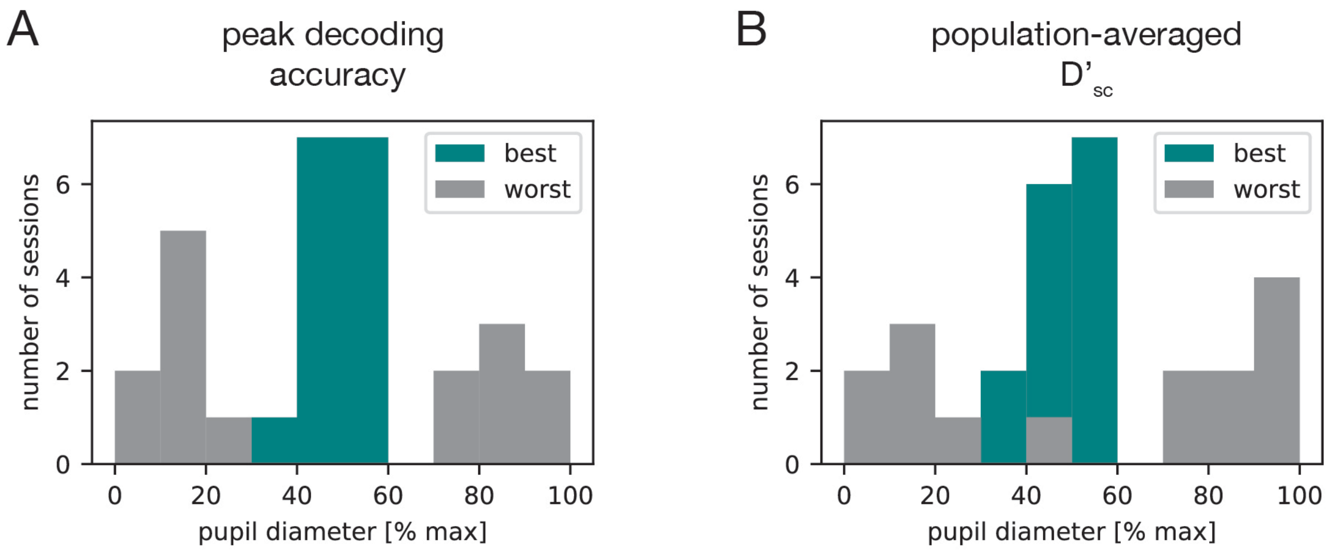

FIG. S5. Pupil diameter distributions corresponding to the best and worst decoding performance (**A**) or population-averaged D'_{sc} (**B**). **(A)** For each session, we determined the pupil decile bin for which the peak decoding accuracy was largest (best decile) or smallest (worst decile). The histogram shows the distribution of the pupil diameter at the middle of the best decile (teal) and worst decile (gray) across all experimental sessions. **(B)** For each session, we determined the pupil decile partition for which the population-averaged D'_{sc} was largest (best decile) or smallest (worst decile). The histogram shows the distribution of the pupil diameter at the middle of the best decile (teal) and worst decile (gray) across all experimental sessions.

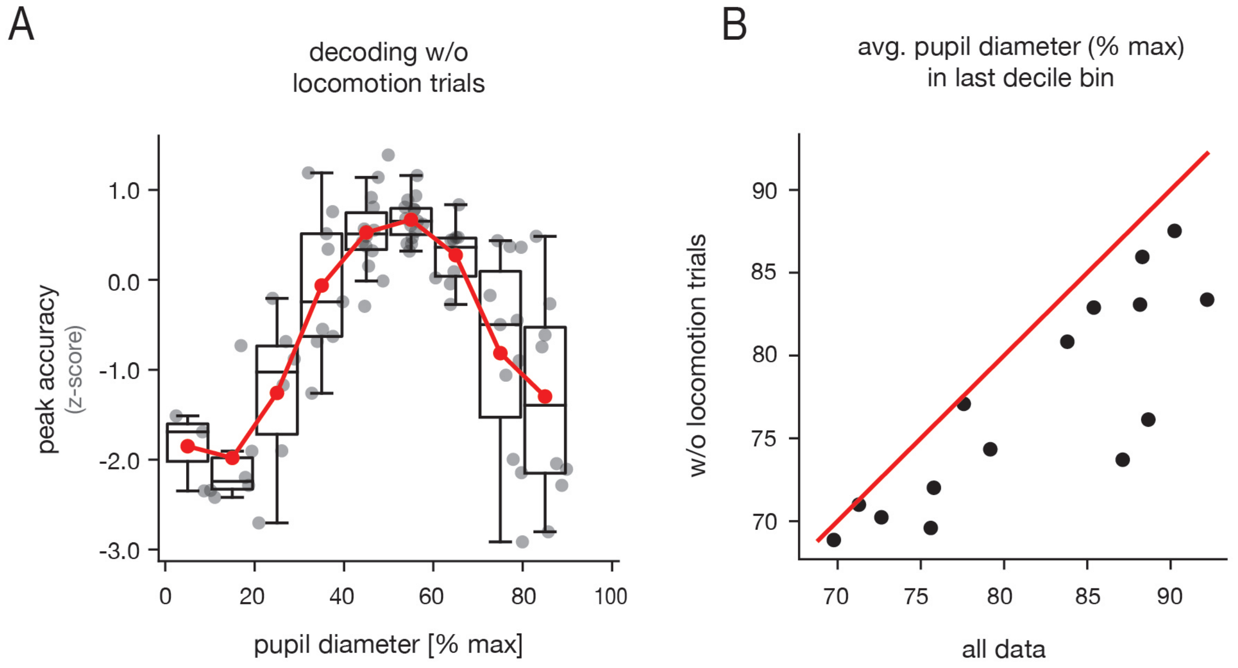

FIG. S6. Population decoding of tone frequency after excluding locomotion trials. **(A)** Peak decoding accuracy (z-scored) *vs.* pupil diameter when locomotion trials are excluded. Within each session, peak accuracy values were z-scored across pupil decile bins. The normalized data was then pooled across all sessions ($n = 15$), and binned by pupil diameter. For each bin, we show individual data points (gray), the mean (red), and corresponding boxplot. The session-averaged decoding performance still follows an inverted-U with pupil diameter, even when locomotion trials are discarded. However, without locomotion trials, large pupil diameters are not as well-sampled and the inverted-U trend is less distinct compared to the case when all data is used (Fig. 2D). See Sec. IV C for methodological details. **(B)** The average pupil diameter of trials in the last decile bin of a session without locomotion trials *vs.* when all data is used. The average pupil diameter is noticeably smaller when locomotion trials are excluded.

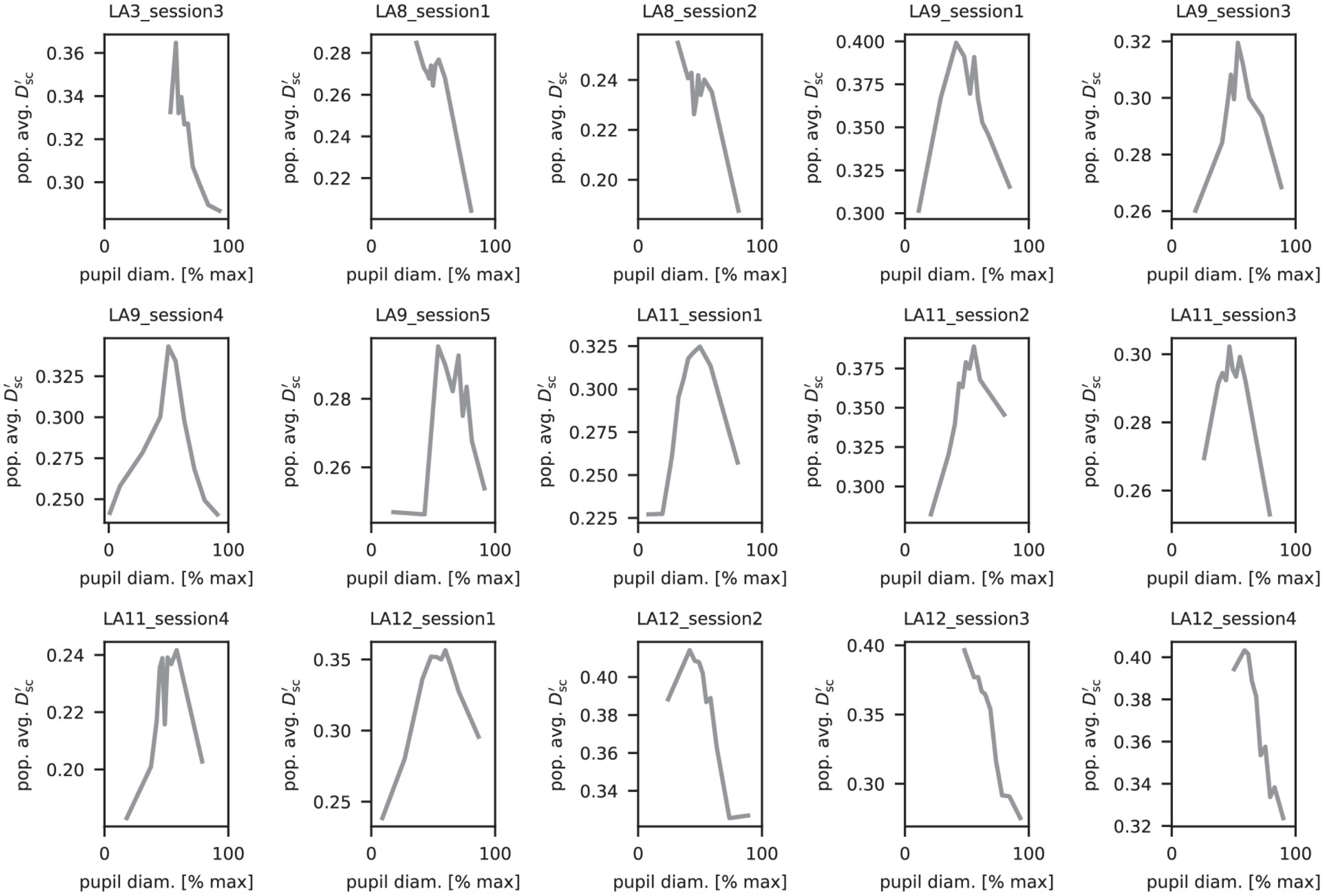

FIG. S7. Population-averaged D'_{sc} *vs.* pupil diameter for all experimental sessions. In most recordings that achieved a broad range of arousal states, the population-averaged D'_{sc} follows an inverted-U relationship with the extent of pupil dilation. For the remainder of sessions, in which only low-to-intermediate or intermediate-to-high diameters were thoroughly sampled, the corresponding upward or downward sloping portions of the curve are apparent (e.g., LA12_session3. See Sec. IV G for methodological details.

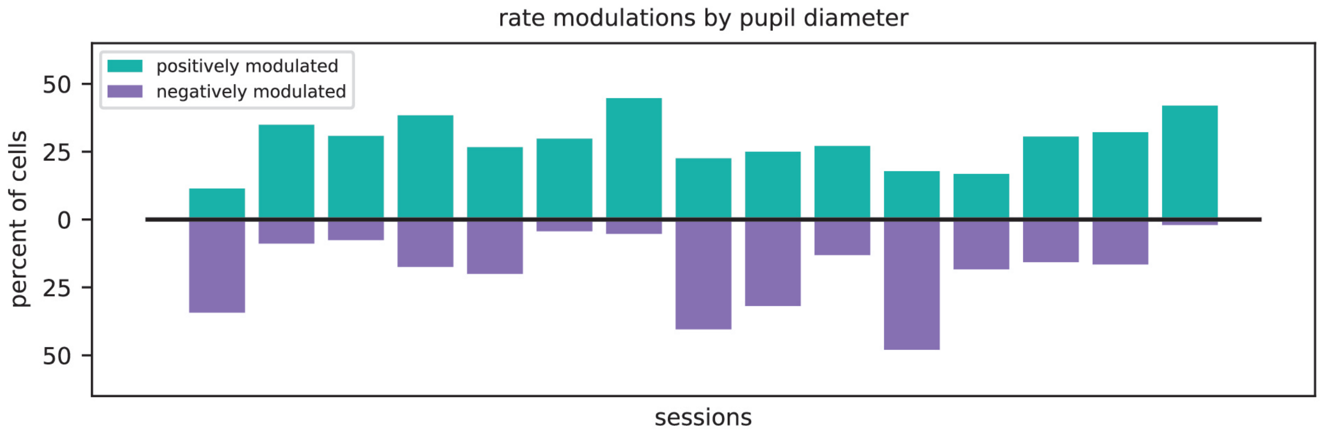

FIG. S8. Fraction of units in each experimental session whose spontaneous firing rate increases (green) or decreases (purple) as a function of pupil diameter. See Sec. IV E for methodological details.

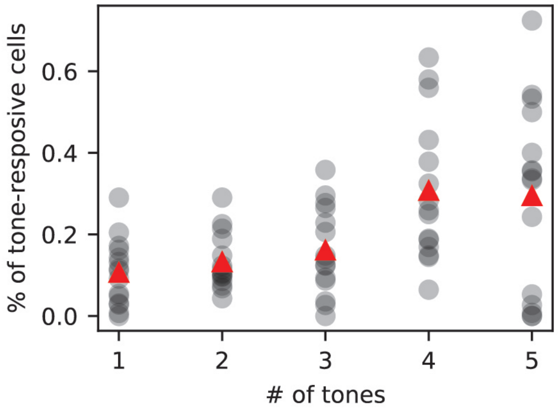

FIG. S9. Fraction of tone-responsive cells that respond to 1, 2, 3, 4, or 5 tones. For a given number of tones, each gray dot corresponds to one experimental session, and the red triangle indicates the mean across sessions. See Sec. IV D for details on determining tone-responsiveness.

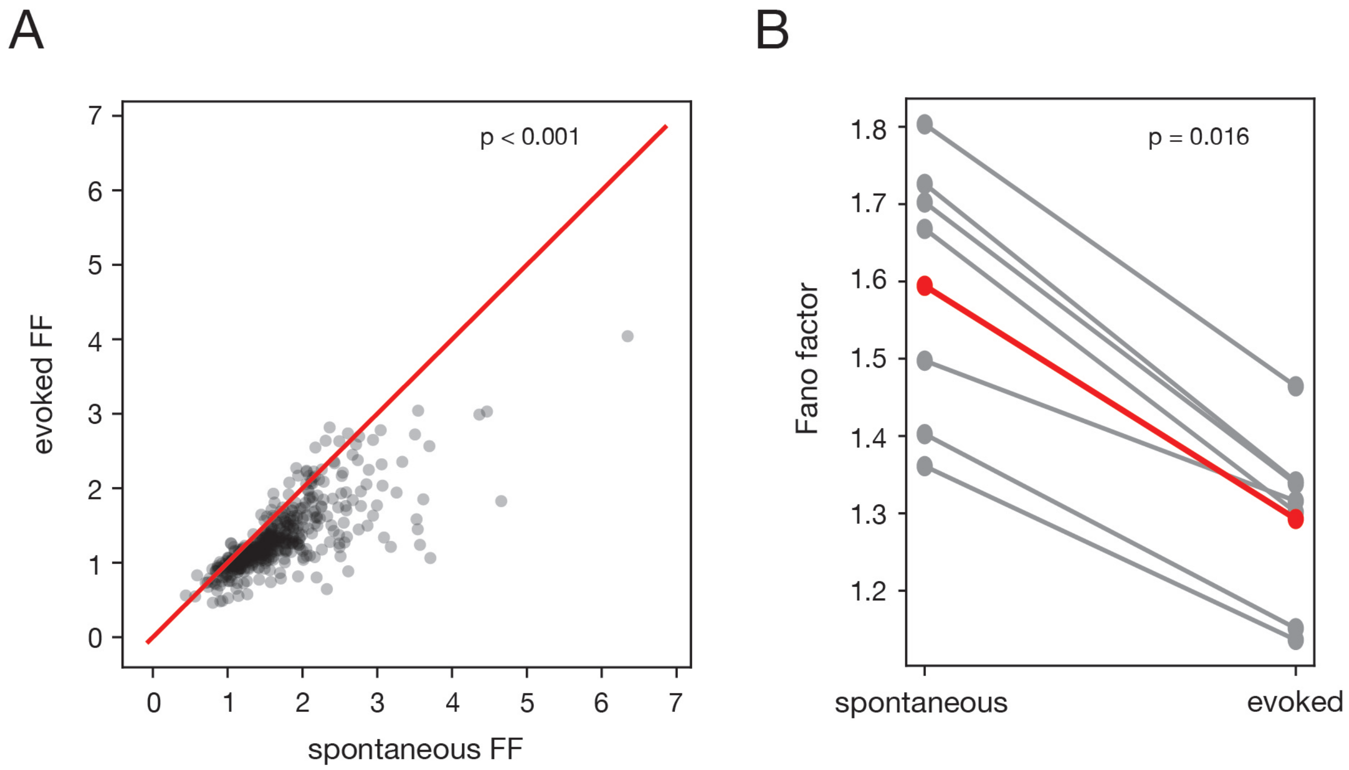

FIG. S10. Stimulus-induced quenching of variability in pupil-aggregated data. To test for overall reductions of neural variability during stimulus presentation, we computed spontaneous and evoked Fano factors using data combined across all pupil diameters in a session (see Sec. IV K 2 for methodological details). **(A)** The pupil-aggregated evoked Fano factor *vs.* the pupil-aggregated spontaneous Fano factor of individual units. The scatter plot contains cells pooled across all sessions that sampled a broad pupil diameter range (i.e., the same sessions analyzed in the pupil-dependent analysis in Fig. 7). There is a significant reduction in the Fano factor in the evoked condition (Wilcoxon signed-rank test, $p < 0.001$, $n = 503$ units), indicating that stimulus presentation leads to a general quenching of neural variability. **(B)** The cell-averaged spontaneous and evoked Fano factor (pupil-aggregated) in each session. There is a significant reduction in the Fano factor in the evoked condition (Wilcoxon signed-rank test, $p = 0.016$, $n = 7$ sessions).

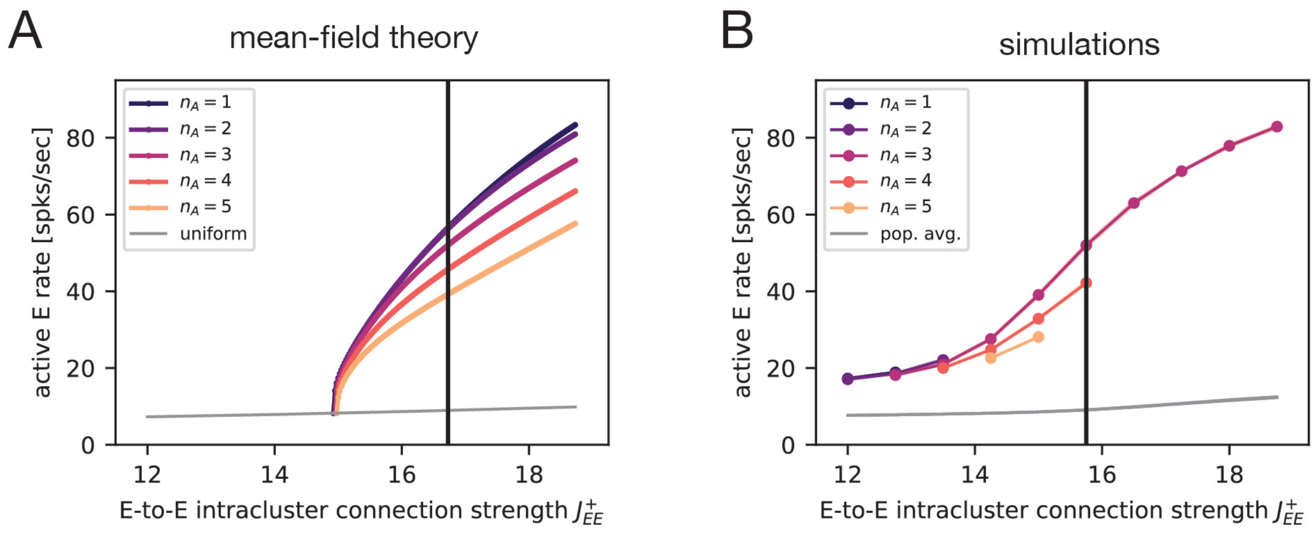

FIG. S11. Strength of E-to-E intracluster coupling controls the onset of cluster states. **(A)** Effect of the E-to-E intracluster coupling strength J_{EE}^{+} on the mean-field solutions of the clustered networks in the absence of the arousal modulation ($\Delta_H^E = 0$). The gray curve shows the rate of the excitatory populations for the solution in which no clusters are active ("uniform" state), and the colored curves show the firing rates of active excitatory clusters for solutions in which $n_A \in \{1, ..., 5\}$ clusters are active ("cluster" states). When J_{EE}^{+} is below a critical value, the mean-field theory has a single, uniform solution (gray), in which all clusters have the same moderate firing rate. As J_{EE}^{+} is increased above a critical value, additional solutions emerge. These cluster states are characterized by $n_A \geq 1$ active clusters with a rate $\nu_{n_A,\uparrow}$. Note that the stability of the solutions is not indicated. **(B)** Effect of the E-to-E intracluster coupling strength J_{EE}^{+} in the simulations. The gray curve shows the average firing rate of all excitatory neurons and the colored curves show the firing rates of active excitatory clusters conditioned on a particular number n_A of active clusters; cluster rates are only plotted for values of n_A that occurred with probability $P(n_A) \geq 0.1$ at a given J_{EE}^{+}. For most values of J_{EE}^{+}, only three clusters are simultaneously active, and the active cluster rate increases significantly with J_{EE}^{+}. Though there are differences between the theory and simulations (specifically, cluster states emerge at lower J_{EE}^{+} in the simulations), the same qualitative behavior is observed in both cases. In panel **A**, the black line corresponds to the value of the E-to-E intracluster coupling strength $J_{EE,\mathrm{mft}}^{+}$ at which the mean-field theory is performed as a function of the Δ_H^E arousal modulation. In panel **B**, the black line corresponds to the value of the E-to-E coupling strength $J_{EE,\mathrm{sim}}^{+}$ that is used in the simulations when studying the impact of Δ_H^E. Note that the mean-field calculations use a larger J_{EE}^{+} than the simulations in order to start with a better match between the mean-field and simulated firing rates when $\Delta_H^E = 0$. See Sec. IV L 3 for details.

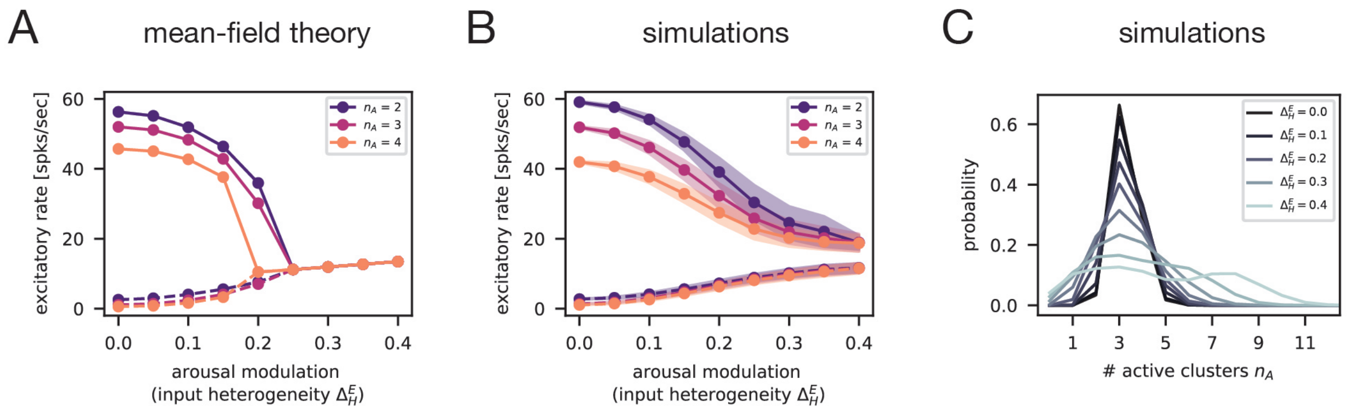

FIG. S12. **(A)** Firing rate of active (solid lines) and inactive (dashed lines) excitatory clusters computed from the mean-field theory as a function of the Δ_H^E arousal modulation. Different colors show the cluster rates for solutions with a particular number n_A of active clusters (see Secs. IV L 2 and IV L 4). As Δ_H^E increases, the distinction between active and inactive cluster rates is decreases; at large values of Δ_H^E, only the uniform state is present. For this analysis, the mean-field calculation was performed with a larger E-to-E intracluster coupling strength than the simulations ($J^+_{EE,\mathrm{mft}} > J^+_{EE,\mathrm{sim}}$); the mean-field intracluster coupling was chosen such that the mean-field and simulated rates approximately matched in the absence of the arousal modulation (Sec. IV L 3). Because the mean-field and simulations are performed at different values of J^+_{EE}, the comparison between the two is only qualitative. **(B)** Firing rate of active (solid lines) and inactive (dashed lines) excitatory clusters as a function of the Δ_H^E arousal modulation in the simulations. Different colors show the cluster rates conditioned on a particular number n_A of active clusters (see Sec. IV H 2). The behavior of the simulations qualitatively matches the mean-field theory, but there is not exact agreement. Lines and shaded areas correspond to the mean ± 1 S.D. over ten network realizations. **(C)** Probability of observing a certain number of active clusters n_A for different values of the Δ_H^E arousal modulation in the simulations (see Sec. IV H 2). Each curve shows the mean over ten network realizations.

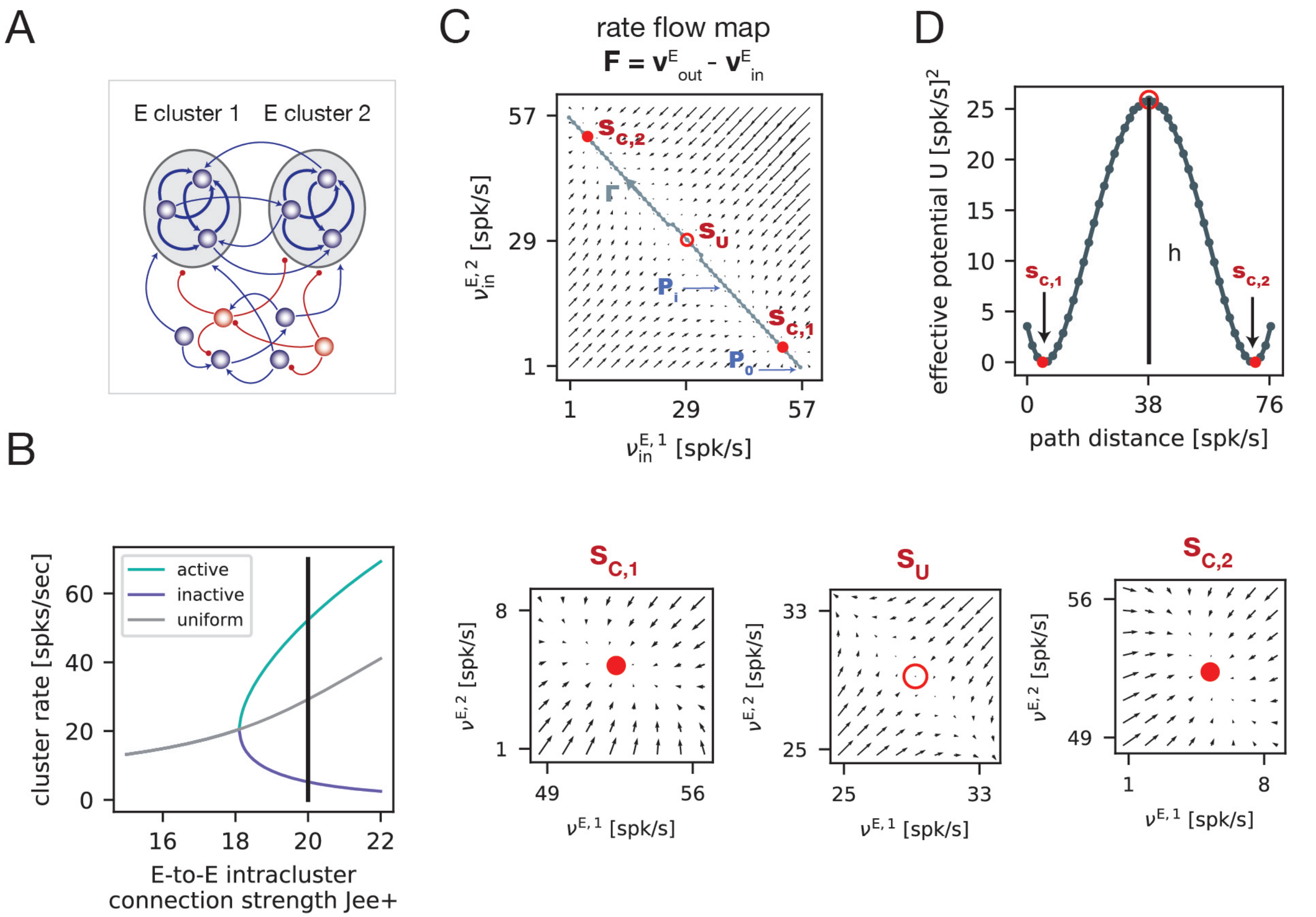

FIG. S13. Details on the mean-field analysis of the 2-cluster circuit. **(A)** Schematic of the 2-cluster network, which contains two excitatory clusters and one background excitatory and inhibitory population (Sec. IV M). **(B)** Effect of the E-to-E intracluster coupling J^+_{EE} on the mean-field solutions of the reduced 2-cluster network (Fig. 5C; Sec. IV M) in the absence of the arousal modulation ($\Delta^E_H = 0$). When J^+_{EE} is below a critical value, the only solution is one in which the two clusters have the same moderate firing rate ("uniform state"). As J^+_{EE} is increased above a critical value, an additional solution emerges in which one cluster is active and the other is inactive ("cluster states"), with rates given by the green and purple curves. Note that the stability of the solutions is not indicated. All analyses of the 2-cluster networks in the main text (Fig. 5D-E) were performed at a fixed E-to-E intracluster coupling $J^+_{EE} = 20$ (black vertical line). **(C)** We studied the dynamics of the 2-cluster network using the effective mean-field theory developed in [1]. To begin, we numerically constructed the rate flow map of the two excitatory clusters, which indicates how the two cluster firing rates will evolve from some initial configuration $\boldsymbol{\nu}^E_{\text{in}}$. To accomplish this, we tiled the $\nu^{E,1}_{\text{in}}$-$\nu^{E,2}_{\text{in}}$ plane with a grid, and at each grid location, we computed the induced output rates $\nu^{E,1}_{\text{out}}$ and $\nu^{E,2}_{\text{in}}$ using the effective theory (Sec. IV M). Here, the rate flow map is visualized by plotting the vector $\mathbf{F} = \boldsymbol{\nu}^E_{\text{out}} - \boldsymbol{\nu}^E_{\text{in}}$ at each grid point. From the rate flow diagram, one can identify the three fixed points from the full mean-field theory in **(B)**, corresponding to the uniform solution (S_U) and the cluster states in which either the first ($S_{C,1}$) or second ($S_{C,2}$) cluster is active. Moreover, the flow map indicates that the uniform solution is unstable, while the two cluster states are attractors. **(D)** To obtain intuition about transitions between the two attractors, we considered a path Γ (gray dotted line in **(C)** connecting the two cluster states $S_{C,1}$ and $S_{C,2}$ through the unstable fixed point S_U. For each point P_i on the path, we computed the line integral $-\int_{\Gamma_{\mathrm{P}_i}} \mathbf{F} \cdot d\boldsymbol{\nu}^E_{\text{in}}$, where Γ_{P_i} denotes the segment of the path from P_0 to P_i. This procedure yields a 1-dimensional effective potential U, which summarizes the cluster dynamics. Specifically, the potential wells correspond to the two attractors $S_{C,1}$ and $S_{C,2}$, and these configurations are separated by a barrier at the unstable fixed point S_U whose height controls the rate of switching between the two cluster states.

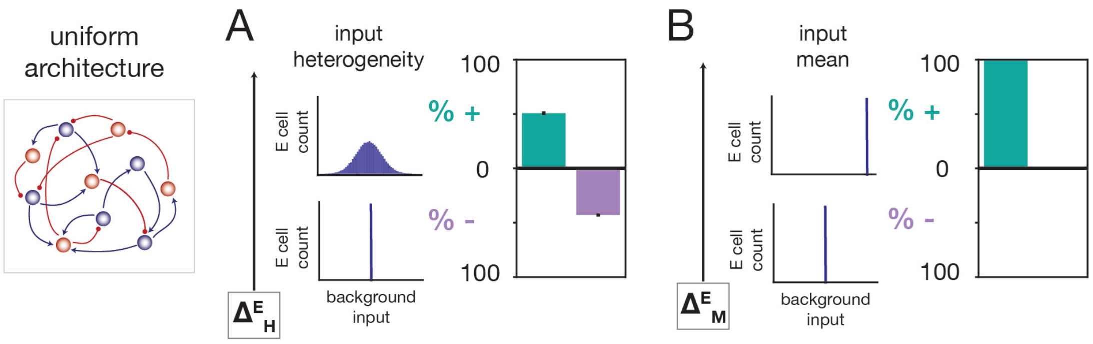

FIG. S14. Effects of the input heterogeneity (Δ_H^E) and input mean (Δ_M^E) arousal modulations on spontaneous firing rates in the uniform networks. **(A)** Fraction of units whose spontaneous firing rate increases (green) or decreases (purple) with Δ_H^E in the uniform networks. **(B)** Fraction of units whose spontaneous firing rate increases (green) or decreases (purple) with Δ_M^E in the uniform networks. Bar heights and error bars correspond to the mean ± the S.D. across 5 network realizations. See Sec. IV F for methodological details.

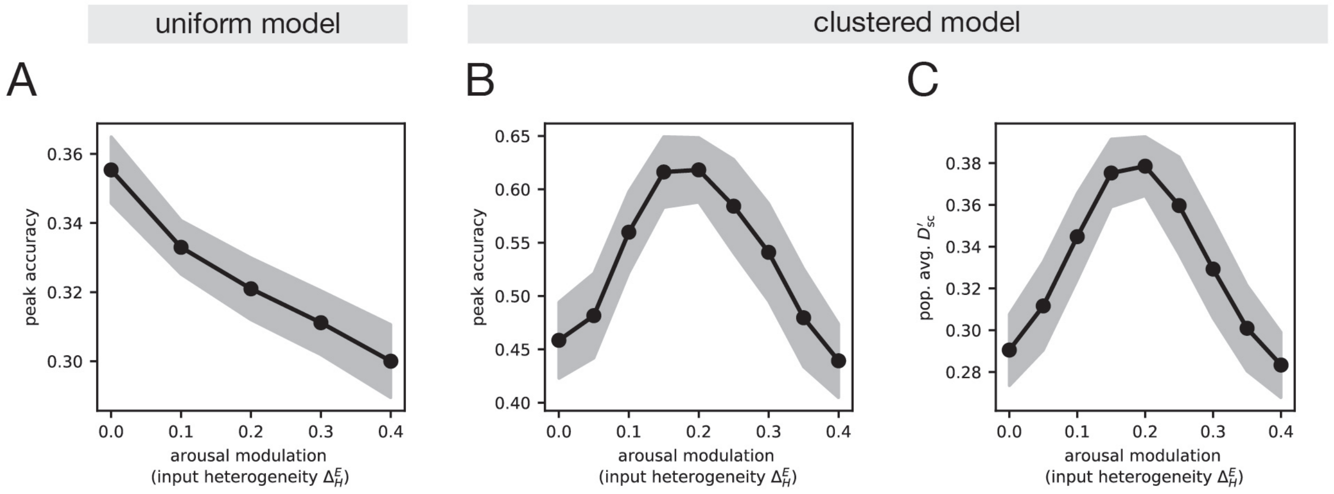

FIG. S15. **(A)** Peak accuracy *vs.* the Δ_H^E arousal modulation in the uniform circuit model. **(B)** Peak accuracy *vs.* the Δ_H^E arousal modulation in the clustered circuit model. **(C)** Population-averaged D'_{sc} *vs.* the Δ_H^E arousal modulation in the clustered circuit model. In all panels, solid lines and shaded areas indicate the mean ± 1 S.D. over 10 network realizations. See Secs. IV C and IV G for methodological details on the decoding analysis and discrminability index D'_{sc}, respectively.

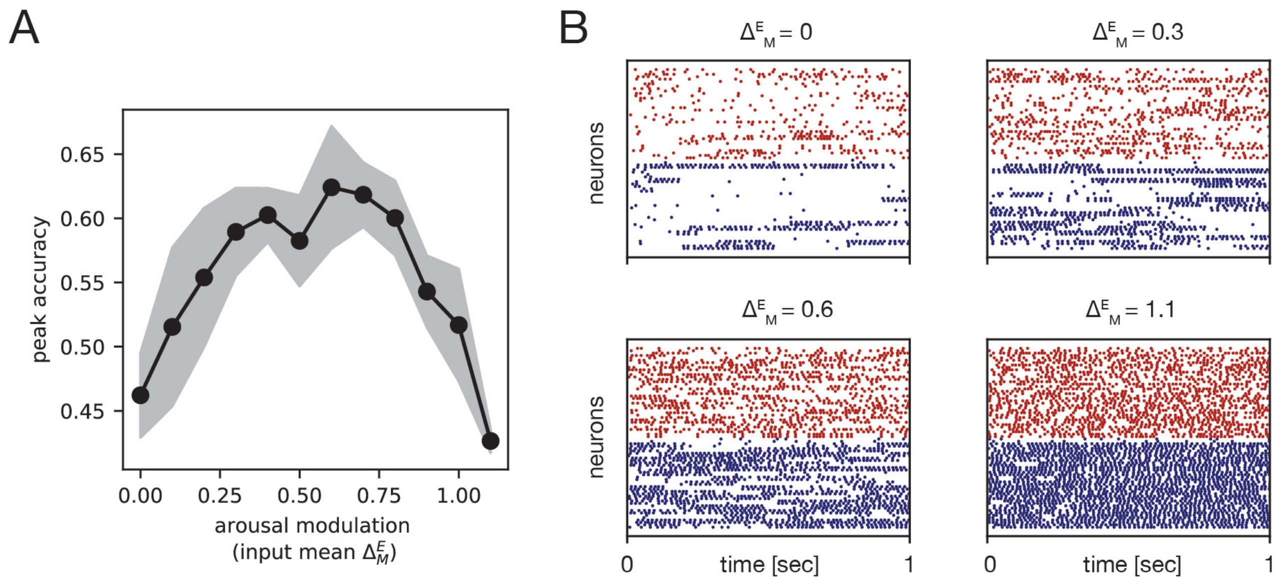

FIG. S16. **(A)** The peak decoding accuracy exhibits an inverted-U relationship with the input mean arousal modulation (Δ_M^E) in the clustered network model. **(B)** Example raster plots showing spontaneous network activity at several values of Δ_M^E. As Δ_M^E increases, more and more clusters become simultaneously active; eventually, the entire network is in a highly-active state.

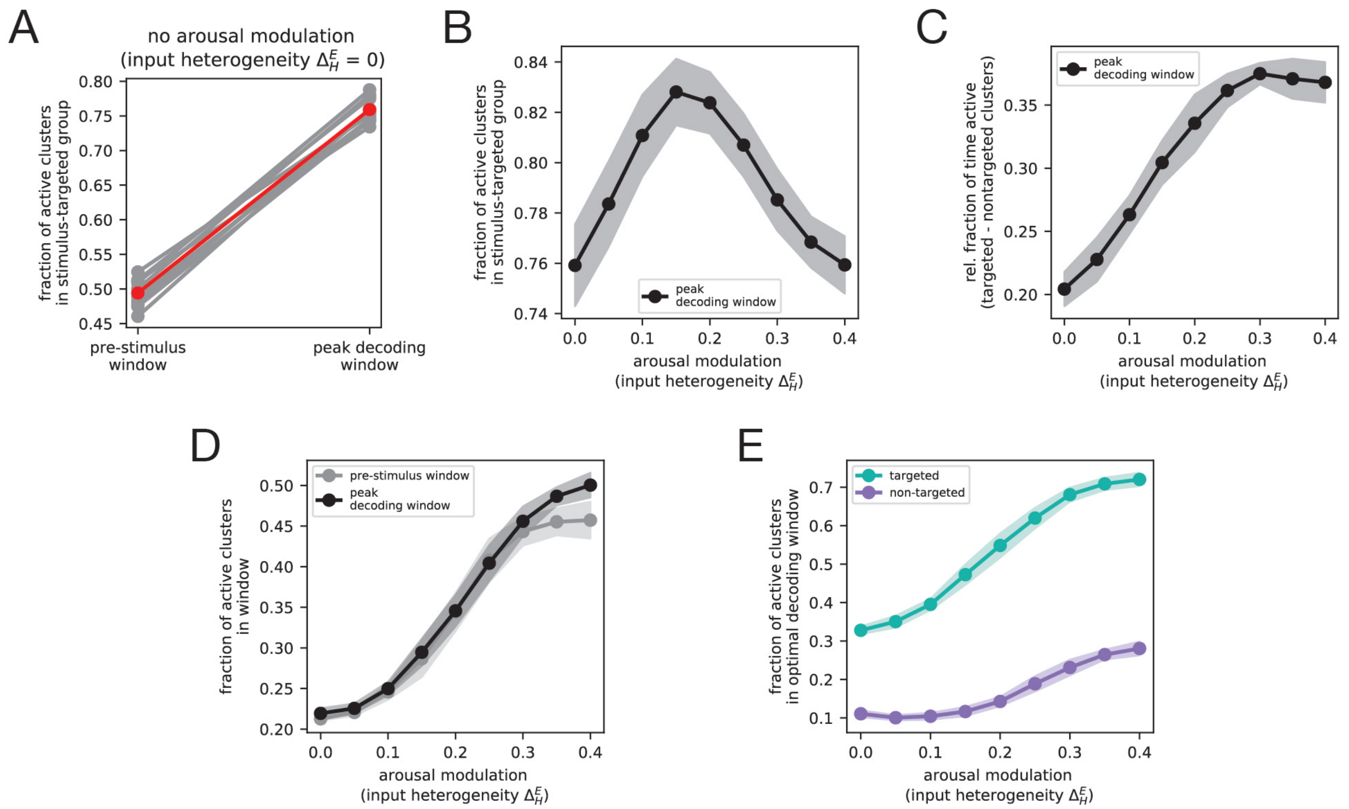

FIG. S17. Additional measures of evoked activity in the clustered model. **(A)** Fraction of active clusters that are part of the stimulus-targeted group ($f_{\uparrow\in\mathrm{T}}$) in the absence of the Δ_H^E arousal modulation. During the pre-stimulus window, the likelihood that an active cluster is part of the targeted group is at chance-level ($f_{\uparrow\in\mathrm{T}}^{\mathrm{spont}} = 50\%$). In contrast, during the peak decoding window, active clusters are significantly more likely to be the stimulated clusters ($f_{\uparrow\in\mathrm{T}}^{\mathrm{evoked}} > 50\%$). **(B)** Fraction of active clusters that are part of the stimulus-targeted group during the peak decoding window ($f_{\uparrow\in\mathrm{T}}^{\mathrm{evoked}}$) as a function of the Δ_H^E arousal modulation. For all Δ_H^E, $f_{\uparrow\in\mathrm{T}}^{\mathrm{evoked}}$ is well above chance levels, indicating that stimuli consistently bias the activation of targeted clusters. Moreover, $f_{\uparrow\in\mathrm{T}}^{\mathrm{evoked}}$ is maximized at intermediate Δ_H^E; in this regime, the transient activation of a cluster is most-strongly related to whether or not that cluster was stimulated. (C) The fraction of time that targeted clusters are active during the peak decoding window relative to nontargeted ones ($\Delta\tilde{\tau}_{N_\uparrow,T_\uparrow}$) as a function of the Δ_H^E arousal modulation. As Δ_H^E increases, stimulated clusters spend more time activated than non-stimulated ones. **(D)** The fraction of all clusters that remain activated for at least 25 ms during the pre-stimulus window ($f_\uparrow^{\mathrm{spont}}$, light gray) or the peak decoding window ($f_\uparrow^{\mathrm{evoked}}$, black) as a function of the Δ_H^E arousal modulation. Both quantities increase with Δ_H^E. **(E)** The fraction of targeted ($f_{T\uparrow}^{\mathrm{evoked}}$) and non-targeted ($f_{N\uparrow}^{\mathrm{evoked}}$) clusters that remain activated for at least 25 ms during the peak decoding window as a function of the Δ_H^E arousal modulation. At moderate Δ_H^E, the increase in $f_{T\uparrow}^{\mathrm{evoked}}$ is driven both by the overall increase in the number of clusters that become activated within a fixed time window (panel **C**) and the increase in the likelihood that active clusters are part of the stimulated subset (panel **B**). The further increase in $f_{T\uparrow}^{\mathrm{evoked}}$ at large Δ_H^E is driven by the former of those two effects. See Sec. IV I for details on how each quantity was computed.

[1] Massimo Mascaro and Daniel J Amit. Effective neural response function for collective population states. *Network: Computation in Neural Systems*, 10(4):351–373, 1999.
